# Inhibition of Phenylpropanoid Biosynthesis in *Artemisia annua* L.: A Novel Approach to Reduce Oxidative Browning in Plant Tissue Culture

**DOI:** 10.1371/journal.pone.0076802

**Published:** 2013-10-07

**Authors:** Andrew Maxwell Phineas Jones, Praveen Kumar Saxena

**Affiliations:** Gosling Research Institute for Plant Preservation, Department of Plant Agriculture, University of Guelph, Guelph, Ontario, Canada; Key Laboratory of Horticultural Plant Biology (MOE), China

## Abstract

Oxidative browning is a common and often severe problem in plant tissue culture systems caused by the accumulation and oxidation of phenolic compounds. The current study was conducted to investigate a novel preventative approach to address this problem by inhibiting the activity of the phenylalanine ammonia lyase enzyme (PAL), thereby reducing the biosynthesis of phenolic compounds. This was accomplished by incorporating 2-aminoindane-2-phosphonic acid (AIP), a competitive PAL inhibitor, into culture media of *Artemisia annua* as a model system. Addition of AIP into culture media resulted in significant reductions in visual tissue browning, a reduction in total phenol content, as well as absorbance and autoflourescence of tissue extracts. Reduced tissue browning was accompanied with a significant increase in growth on cytokinin based medium. Microscopic observations demonstrated that phenolic compounds accumulated in discrete cells and that these cells were more prevalent in brown tissue. These cells were highly plasmolyzed and often ruptured during examination, demonstrating a mechanism in which phenolics are released into media in this system. These data indicate that inhibiting phenylpropanoid biosynthesis with AIP is an effective approach to reduce tissue browning in *A. annua*. Additional experiments with *Ulmus americana* and *Acer saccharum* indicate this approach is effective in many species and it could have a wide application in systems where oxidative browning restricts the development of biotechnologies.

## Introduction

Oxidative browning is a common problem in plant tissue culture; resulting in reduced growth [1,2], lower rates of regeneration or recalcitrance [3–5], and can ultimately lead to cell/tissue/plant death [1,4,6–8]. The prevalence of browning varies among species, cultivars, and the physiological state of the plant/tissue but in many cases severely restricts our ability to manipulate plant growth and development. The underlying cause of tissue browning is the accumulation and subsequent oxidation of phenolic compounds in the tissue and culture media. While phenolic compounds are generally present in healthy plant tissues and can accumulate in specialized cell types [9], they are produced in greater abundance and/or released as a defense response, especially following tissue wounding or stress [9,10]. The majority of tissue culture protocols involve wounding the material in order to remove explants and culturing them in potentially stressful environments; often eliciting the production and release of phenolic compounds. As a result, this natural defense response can lead to the accumulation of toxic compounds that ultimately damage or kill plant cells and tissues.

Due to the ubiquitous nature and severe consequences of tissue browning, a substantial amount of research has gone into developing methods to prevent and/or ameliorate it [1,6,11–16] several advances have been made towards reducing oxidative browning by altering environmental conditions used in tissue culture. For example, tissues cultured in the dark often display lower levels of browning than those grown in the light [1,17,18]. Changing the basic media composition and the type/concentration of plant growth regulators can also reduce the degree of browning. A more targeted approach of pre-treating explants and/or amending culture media with compounds specifically selected to reduce tissue browning is also often employed [6]. Most of these treatments/amendments can be divided into two general categories: 1) antioxidants such as ascorbic acid, melatonin, or citric acid, that reduce oxidative stress and prevent oxidation of phenolic compounds [2–2,6) adsorbants that bind phenolic compounds rendering them less toxic such as activated charcoal or PVPP [2,16]. These approaches are often combined with frequent sub-cultures to reduce exposure [19], although in some species frequent subculture exacerbates the problem, presumably by further stressing the explant [20]. The onset of browning remains unpredictable and can occur even in plants that are amenable to culture due to genotypic variation and differences in microenvironments of plant tissue culture.

While the aforementioned approaches to reduce browning have improved several culture systems, the problem persists in many species and new methods are needed to address this fundamental challenge in plant tissue culture. Since tissue browning results from the accumulation and subsequent oxidation of phenolic compounds, it is intimately linked to phenylalanine ammonia lyase (PAL) activity [3,6]. PAL is the first dedicated enzyme in the phenylpropanoid pathway and converts phenylalanine into trans-cinnamic acid, providing the substrate for further synthesis of phenolic compounds [10]. Numerous studies have found that PAL activity increases prior to, or during tissue browning, and that methods targeted at reducing browning often lower PAL activity [1,3,8]. As such, focussing on methods that reduce PAL activity is a logical approach to develop novel methods to reduce oxidative browning and improve culture techniques.

The phenylalanine structural analog, 2-aminoindane-2-phosphonic acid (AIP), is a well documented competitive inhibitor of PAL both in vitro and in vivo, and provides an effective means to prevent the synthesis of phenolic compounds in a variety of systems [21–33]. Inhibition of PAL through the use of AIP has the potential to significantly reduce the biosynthesis of phenolic compounds, and could represent a novel approach to controlling oxidative browning in plant tissue culture. This approach has been successful in preventing post harvest browning in lettuce [34], and previous qualitative observations indicate that it reduces browning in a two phase callus/suspension culture of American elm [35]. However, to date it has not been empirically tested as a method to reduce browning in plant tissue culture. The current study was conducted to evaluate the application of AIP to reduce tissue browning using *Artemisia annua* callus cultures, which often exhibit tissue browning [36–39], as a model system. Incorporation of AIP into the culture media significantly reduced phenolic content of the tissue, resulted in a stark reduction in visual browning, and generally increased tissue growth. Preliminary experiments were also conducted using sugar maple (*Acer saccharum*) and American elm (*Ulmus americana*) callus cultures to evaluate the efficacy of this approach in reducing browning in other species.

## Materials and Methods

### Plant growth

Seeds of *Artemisia annua* hybrid 1209 were kindly provided by East-West Seed (Nonthaburi, Thailand). The seeds were surface sterilized in 10% commercial bleach (6.25% sodium hypochlorite; Chlorox) with approximately 0.1% tween 20 (Fisher Scientific, Canada) for ten minutes, followed by three rinses in sterile distilled water. The seeds were then transferred into GA7 culture vessels (Magenta, Chicago, USA) each containing about 20 ml of MS basal medium. The MS medium was comprised of MS salts and vitamins [40] (Phytotechnology; Shawnee Mission, USA), 30 g/l sucrose, and 7g/l agar (Fisher Scientific, Canada). The pH of the medium was adjusted to 5.7 before addition of agar and prior to being autoclaved at 121°C and 21 psi for 20 min. The cultures were maintained in a growth room at 24°C ± 2°C under a 16 h photoperiod (40 µmol/m^2^/s) provided by cool-white fluorescent lamps (Philips Canada, Scarborough, ON).

### Plant tissue culture

*Artemisia annua* explants were removed from 5 day old seedlings prior to the emergence of the first true leaves. The two cotyledons and the hypocotyl from each seedling were separated and cultured in separate Petri dishes (50 X 15 mm; Fisher Scientific, Canada) containing approximately 10 ml of culture medium for a total of three explants per plate. The basic media tested were modified versions of those previously optimized to induce and maintain undifferentiated tissue in *Artemisia annua* [41], and were comprised of MS salt and vitamins [40], 30 g/l sucrose, 7 g/l agar, and either 4.5 µM 2,4-D (Sigma-Aldrich, Canada) or 11 µM BA (Sigma-Aldrich, Canada) in combination with 2.7 µM NAA (Sigma-Aldrich, Canada). A dose response of AIP was conducted using the 4.5 µM 2,4-D medium supplemented with 1, 10, or 100 µM AIP, which was synthesized (SV ChemBioTech, Inc, Edmonton, AB) as described previously [21]. For more detailed study, media supplemented with 4.5 µM 2,4-D or 11 µM BA and 2.7 µM NAA were evaluated with and without the addition of 100 µM AIP. All media were adjusted to a pH of 5.7 prior to adding agar and being autoclaved at 121°C and 21 psi for 20 min. The cultures were maintained at 24°C ± 2°C in the dark.

For sugar maple (*Acer saccharum*) and American elm (*Ulmus americana*) studies, callus was obtained from materials maintained at the Gosling Research Institute for Plant Preservation. In both cases, callus was originally derived from mature trees and was maintained on basal media comprised of MS salt and vitamins [40], 30 g/l sucrose, 7 g/l agar, 5 µM BA (Sigma-Aldrich, Canada), and 1 µM NAA (Sigma-Aldrich, Canada). Callus explants were transferred onto the same media with and without the addition of 1 mM AIP. Cultures were grown for 6 weeks before being visually assessed for browning.

### Sample preparation and extraction

The callus from each *A. annua* culture plate was weighed, transferred into a 15 ml centrifuge tube (Fisher Scientific, Canada), and flash frozen in liquid nitrogen. Samples were then lyophilized for at least 24 hours (Freezone 4.5; Labconco, Kansas city, USA) until dry. Each sample was evaluated at the same time by a single observer for visual tissue browning using a hedonic scale ranging from 0 to 10; 0 being no observable browning and 10 representing dark brown/black tissue. Samples were finely ground and approximately 10 mg of each sample was transferred into a 1.5 ml micro-centrifuge tube (Fisher Scientific, Canada). An aliquot of extraction solvent (1:1:1 water: methanol: acetone) was added to each tube such that the tissue to solvent ratio was 1:10. The tubes were then vortexed and placed in a sonicating water bath (Branson 3510, Danbury, USA) for three hours. The tubes were then removed and centrifuged for 5 minutes at 21.1*g*. The supernatant from each sample was then transferred into a new micro-centrifuge tube.

### Extract analysis

Total phenols were estimated using a modified Folin- Ciocalteu assay using gallic acid (Sigma-Aldrich, Canada) as the standard [42]. In brief, 10 µl aliquots of sample extracts, standards, or sample blanks were added to each well of a 96 well flat bottom microplate (Corning, Corning, USA). To each well a volume of 100 µl of Folin and Ciocalteu phenol reagent (MP Biomedicals, USA) was added and the plate was incubated for 5 minutes before adding 80 µl of aqueous 0.25 M Na _2_CO_3_. The plate was then incubated in the dark for 1 hour before the absorbances at 740 nm were measured with a Synergy H1 microplate reader (Biotek, Winooski, USA) and all sample and standard readings were corrected with blanks.

Absorbance at 340 nm was measured as a proxy for measuring tissue browning as previously described [34]. Ferulic and chlorogenic acids (Sigma-Aldrich, Canada) were used as standards to estimate the total phenolic content of the extracts. Aliquots of 10 µl from each sample, standard concentration, or sample blank, were added to wells of a 96 well flat bottom microplate (Corning, Corning, USA). Another 190 µl of the extraction solvent was added to each of the wells. The absorbance from each well was measured with a Synergy H1 microplate reader (Biotek, Winooski, USA) and all sample and standard readings were corrected with the blanks. The absorbance spectrum of each sample, standard, and blank, was also read using the spectral scan function between 300-700 nm at 5 nm increments.

**Figure 1:**
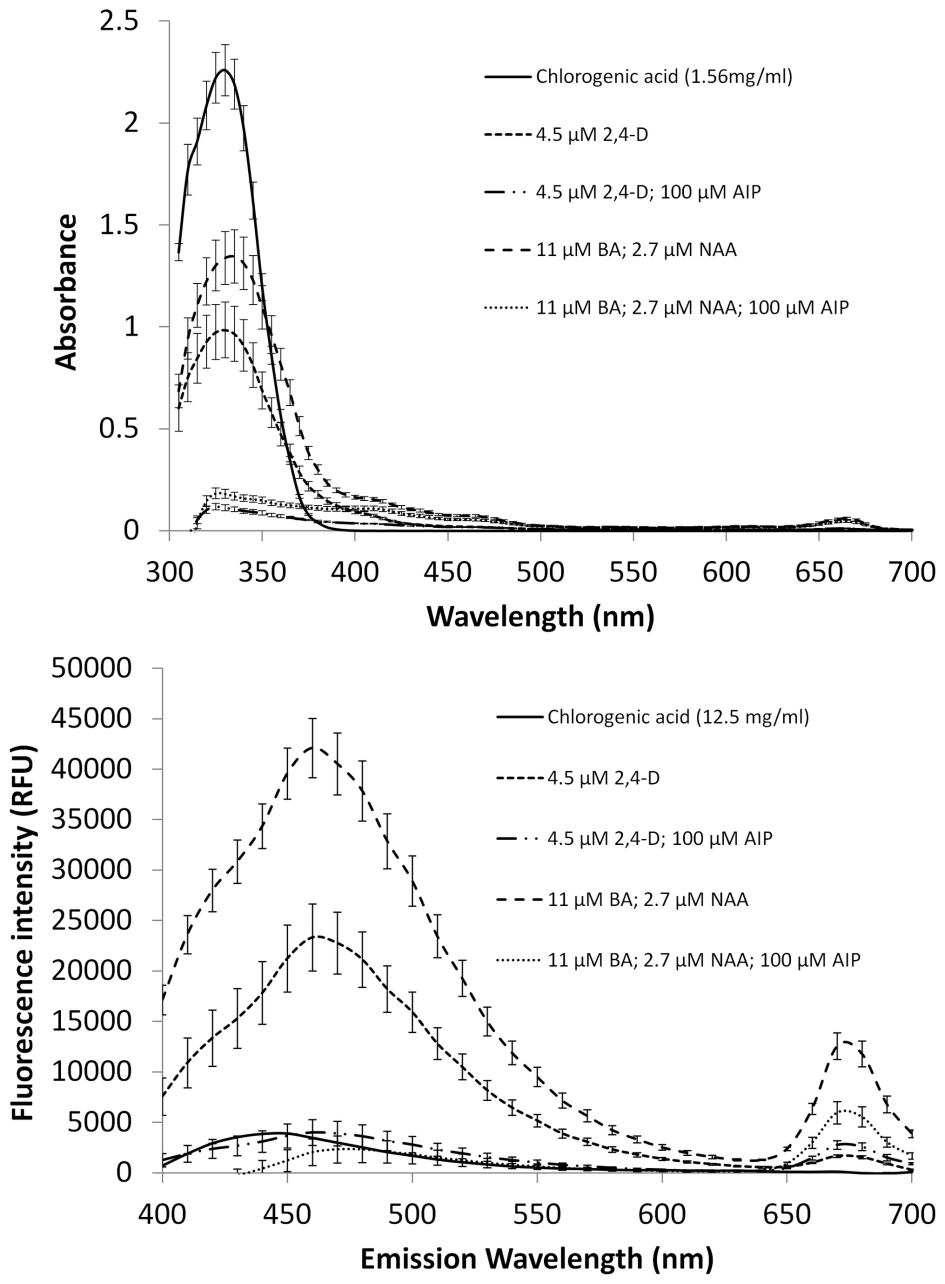
Absorbance and Fluorescence spectra (360 nm excitation) of extracts from *Artemisia annua* callus cultured on 11µM BA/2.7 µM or 4.5 µM 2,4-D based media with and without addition of 100 µM AIP compared to that of chlorogenic acid. Points represent the mean of seven biological replicates with bars indicating the standard errors of the means.

The autofluorescent properties of the extracts were evaluated for all of the samples, as well as ferulic acid, chlorogenic acid, cinnamic acid (Sigma-Aldrich, Canada), and caffeic acid (Sigma-Aldrich, Canada) as potential standards. Ten microlitre aliquots of each sample, standard, and blank, were combined with 190 µl of extraction solvent in a 96 well black microplate (Corning, Corning, USA). The optimal excitation wavelength was first optimized using a Synergy H1 microplate reader (Biotek, Winooski, USA). This was accomplished with a sample extract using the spectral scan function to evaluate excitation wavelengths from 300-400 nm with a fixed emission wavelength of 460 nm based on previous experience with phenolic based blue-green autofluorescence of plants [43]. The optimal emission wavelength was determined using a fixed excitation wavelength of 360 nm based on the previous optimization step to conduct a spectral scan between 400-700 nm with 5 nm increments. This procedure was done for all samples, extracts, and blacks to produce the fluorescence spectra shown in Figure 1. Using the optimized excitation/emission wavelengths of 360 nm and 450 nm respectively, endpoint measurements were taken for all of the wells. All endpoint and spectral scan values were corrected with the average readings from the solvent blanks.

### Microscopic evaluation

Autofluorescence of the samples were observed using an inverted epi-fluorescent microscope (Axiovert 200; Carl Zeiss Canada Ltd., Canada) with a wide UV excitation and longpass emission filter set (Chroma, Bellows Falls, VT). Cell viability was observed with the same microscope after the cells were incubated for 10 minutes in the dark in liquid media of the same composition (without agar) with the addition of 60 µl/ml Fluorescein diacetate (2mg/ml; Sigma-Aldrich, Canada) prepared in acetone. Viable cells were visualized using a Fitc/Bodipy/Fluo3/Dio filter set (Chroma, Bellows Falls, VT). Images were acquired using a PowerShot G12 digital camera (Canon, Mississauga, ON) mounted on the microscope in manual mode using uniform camera settings. Autofluorescence was further observed using an upright Leica DM 6000B confocal laser scanning microscope (Leica, Wetzlar, Germany) connected to a Leica TCS SP5 system. Emissions between 430-480 nm were collected using a radius 405 nm laser set at 20% power for excitation. The pinhole was set at 60 µm and each image was captured using the average of three passes of the image. All gain settings were first optimized to control tissues and the same settings were used to observe cells grown in the presence of AIP in order to provide standardized comparisons.

### Experimental design and statistical analysis

The experiment was arranged in a completely randomized design with seven replicate plates containing 3 explants per plate for each treatment, and the experiment was conducted twice. All statistical analyses were conducted using JMP 10 (SAS institute, NY) with a p-value of 0.05. An analysis of variance was conducted to determine the significance of the model for each trait that was evaluated. Means separations were conducted using Tukey’s honest significance test. Correlations were conducted using a multivariate analysis to determine if there were significant correlation values and determine the R values. Regression analyses were conducted for all standard curves and between various assays and visual tissue browning scores to determine there were significant predictive relationships and what the coefficients of determination were.

## Results

### *A. annua* tissue growth

**Figure 2:**
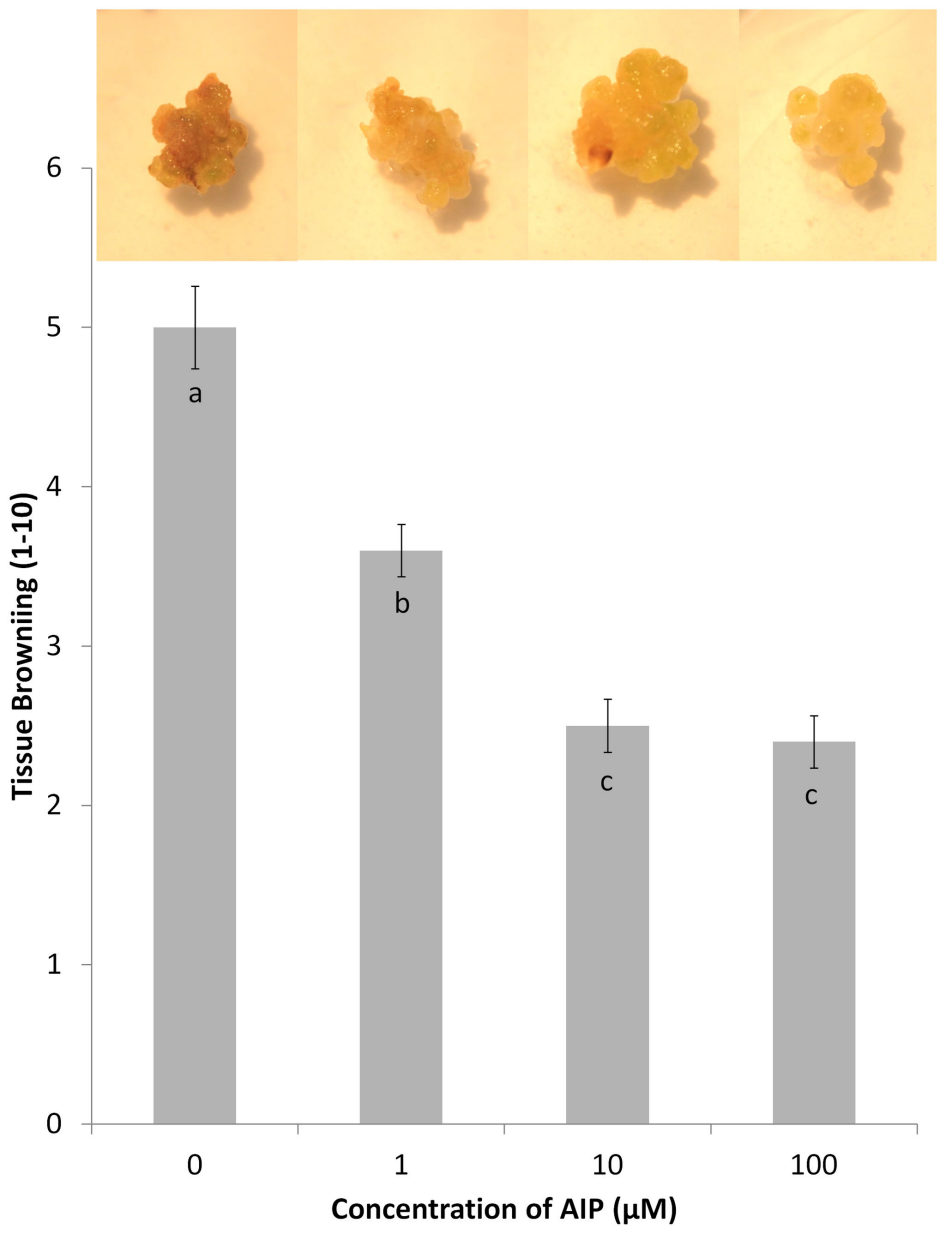
Visual browning scores (1-10) of *Artemisia annua* callus cultured on 4.5 µM 2,4-D based media supplemented with various levels of 2-aminoindane-2-phosphonic acid. Bars represent the mean of seven replicates plus or minus the standard error of the mean. Images above the bars display callus grown on the corresponding level of AIP.

The dose response of AIP demonstrated that tissue browning declined in a dose dependent manner up to 10 µM AIP. The incorporation of 100 µM AIP produced callus with a similar degree of browning as 10 µM AIP (Figure 2). *Artemisia annua* cotyledons cultured on the four test media (4.5 µM 2,4-D ± 100 µM AIP and 11 µM BA + 2.7 µM NAA ± 100 µM AIP) all produced callus, but there were significant differences in the growth among treatments (Table 1). The fresh weight of callus was significantly higher on 11 µM BA/ 2.7 µM NAA medium (634.2 mg/plate ± SE 186.28), hereon referred to as BA/NAA medium, compared to medium containing 4.5 µM 2,4-D (150.9 mg/plate ± SE 17.31), hereon referred to as 2,4-D medium. This pattern was also observed in measurements of dry weights, with average callus weights of 70.4 mg/plate ± SE 20.67 and 10.0 mg/plate ± SE 1.14 on BA/NAA and 2,4-D media, respectively. Callus produced on both media exhibited a similar degree of tissue browning based on a hedonic scale (2,4-D medium: 4.5 ± SE 0.22; BA/NAA medium: 4.0 ± SE 0.58), but it was less obvious on BA/NAA medium due to the presence of chlorophyll and the sporadic occurrence of shoot organogenesis.

**Table 1:** Growth and analysis of *Artemisia annua* callus grown for 5 weeks in the dark.

	Callus growth (mg/plate)	Visual Browning (0-10)	Total phenolic content			Fluorescence Intensity (340nm/460nm)
			Folin assay	Absorption at 340nm		
			galic acid equivilents	Ferulic acid equivilents	Chlorogenic acid equivilents	
Cotyledon callus - Dry weight basis			mg/g	mg/g	mg/g	RFUs
4.5 µM 2,4-D	10.0±1.1c	4.5±0.22a	4.4±0.4a	5.3±0.8b	7.1±1.1b	32831±4846b
4.5 µM 2,4-D; 100 µM AIP	12.2±2.3c	1.0±0.32b	1.7±0.1b	0.7±0.1c	0.4±0.1c	5783±1500c
11 µM BA; 2.7 µM NAA	70.4±20.7b	4.0±0.58a	4.9±0.4a	7.6±0.7a	10.5±1.1a	50207±6375a
11 µM BA; 2.7 µM NAA; 100 µM AIP	116.3±22.8a	0.6±0.24b	2.1±0.1b	1.0±0.1c	1.0±0.1c	8772±2520c
Cotyledon callus - Fresh weight basis			µg/g	µg/g	µg/g	RFUs
4.5 µM 2,4-D	150.9±17.3c	ND	476±52a	632±100a	859±144a	ND
4.5 µM 2,4-D; 100 µM AIP	182.8±34.2c	ND	151±21c	60±9b	37±8b	ND
11 µM BA; 2.7 µM NAA	634.2±186.3b	ND	325±36b	501±55a	692±78a	ND
11 µM BA; 2.7 µM NAA; 100 µM AIP	1268.9±248.6a	ND	119±22c	68±6b	63±7b	ND

Values represent the mean and standard error of 7 individual Petri plates. Values within each group followed by a different letter are significantly different at 0.05 using a student’s means separation with Tukey’s adjustment.

Addition of 100 µM AIP to BA/NAA medium resulted in nearly a two fold increase in callus growth (1268.9 mg/plate ± SE 248.55) compared to basal BA/NAA medium. While there was about a 20% increase in callus growth observed in explants cultured on 2,4-D medium supplemented with 100 µM AIP (182.8 mg/plate ± SE 34.21) compared to basal 2,4-D medium, this difference was not statistically significant. This was also observed in respect to dry weight with BA/NAA + 100 µM AIP, producing 116.3 mg/plate ± SE 22.78, and 2,4-D + 100 µM AIP medium producing 12.2 mg/plate ± SE 22.78. In both cases, the addition of AIP significantly reduced visual browning scores (2,4-D + 100 µM AIP medium: 1.0 ± SE 0.32; BA/NAA + 100 µM AIP medium: 0.6 ± SE 0.24). Other than the reduction in tissue browning, the calli were morphologically similar to the controls, including the accumulation of chlorophyll in the BA/NAA + AIP induced callus and the occurrence of shoot organogenesis.

### Phenolic content – Folin assay

The co-efficient of determination of a four point standard curve using gallic acid was 0.9974 between 125 µg/ml and 1000 µg/ml with an average RSD of 2.9%, and all samples used in the analysis fell within this linear range. Callus grown on 2,4-D medium contained a similar amount of total phenols (4.4 mg/g ± SE 0.39) as callus that developed on BA/NAA medium (4.9 mg/g ± SE 0.43) on a dry weight basis. However, on a fresh weight basis, callus grown on 2,4-D medium contained significantly more total phenols (476 µg/g ± SE 52.6) than callus developed on BA/NAA medium (325 µg/g ± SE 35.9). The addition of AIP into either media resulted in significant reductions in total phenol content on both a dry weight (2,4-D + 100 µM AIP medium: 1.7 mg/g ± SE 0.06; BA/NAA + 100 µM AIP medium: 2.1 ± SE 0.08) and a fresh weight basis (2,4-D + 100 µM AIP medium: 151 µg/g ± SE 21.3; BA/NAA + 100 µM AIP medium: 119 µg/g ± SE 21.9). The total phenol content in callus cultured on both media supplemented with AIP was statistically similar to one another on a dry or fresh weight basis.

### Phenolic content – Absorbance at 340 nm

The co-efficient of determination of a six point standard curve using ferulic acid was 0.9996 between 49 µg/ml and 1563 µg/ml with an average RSD of 2.7%, and all samples used in the analysis fell within this linear range. Based on this method of analysis, callus grown on BA/NAA medium contained significantly more total phenols (7.6 mg/g ± SE 0.73) than callus developed on 2,4-D medium (5.3 mg/g ± SE 0.76) on a dry weight basis. However, on a fresh weight basis, callus grown on 2,4-D medium contained a similar amount of total phenols (632 µg/g ± SE 100.0) as callus that developed on BA/NAA medium (501 µg/g ± SE 55.2). The addition of AIP into either media resulted in significant reductions in total phenol content on dry weight (2,4-D + 100 µM AIP medium: 0.7 mg/g ± SE 0.06; BA/NAA + 100 µM AIP medium: 1.0 ± SE 0.09) and fresh weight basis (2,4-D + 100 µM AIP medium: 60 µg/g ± SE 8.7; BA/NAA + 100 µM AIP medium: 68 µg/g ± SE 6.3). The total phenol content in callus cultured on media supplemented with AIP was statistically similar to one another on a dry or fresh weight basis.

The co-efficient of determination of a six point standard curve using chlorogenic acid was 0.9999 between 49 µg/ml and 1563 µg/ml with an average RSD of 1.2%, and all samples used in the analysis fell within this linear range. The statistical comparisons among the various samples were the same as what was observed using ferulic acid as the standard, but absolute values were different due to the different absorptive properties of chlorogenic and ferulic acid (Table 1). The absorbance spectra of the samples agreed with the readings taken at 340nm (Figure 1a). Extracts of callus gown without AIP produced a main peak with a maximum absorbance around 340 nm, similar to the peak produced by chlorogenic acid, while this peak was almost absent from the extracts of callus grown on AIP.

### Phenolic content – Fluorescence

The relationship between the concentration and fluorescence intensity of ferulic acid was best fit by the following equation: y = 2077.7x^0.3037^ between 49-6250 µg/ml with a co-efficient of determination of 0.9915 and an average RSD of 2.7%. Above this concentration, the fluorescence intensity plateaued. Chlorogenic acid produced a similar response but plateaued at a lower concentration. The relationship of a seven point curve between 49-3125 µg/ml for chlorogenic acid was best described with the following equation: y = 1895.5x^0.2486^, giving a co-efficient of determination of 0.9928 and an average RSD of 1.2%. Caffeic and cinnamic acids did not fluoresce using a 360 nm excitation wavelength (data not shown). The majority of sample extracts produced fluorescence emissions much higher than either standard.

A dilution series of the most fluorescent sample extract responded in a linear fashion using an 8 point curve ranging from a 1/256 dilution to the full strength extract, producing a co-efficient of determination of 0.9949. Callus grown on BA/NAA medium produced significantly higher fluorescence readings (50207RFUs ± SE 6374.6) than callus from 2,4-D medium (32831RFUs ± SE 4846.1). Callus produced on either media supplemented with 100 µM AIP produced similar readings, significantly lower than without AIP (2,4-D + 100 µM AIP medium: 5783RFUs ± SE 1500.3; BA/NAA + 100 µM AIP medium: 8772RFUs ± SE 2519.5).

Spectral scans of the fluorescence emissions emitted from the samples using an excitation wavelength of 360 nm were in agreement with the endpoint scans (Figure 1b). All sample extracts produced a second peak with a maximum intensity at about 775nm, but this was more pronounced in the BA/NAA media. The fluorescence peak produced by chlorogenic and ferulic acid were similar to one another but their peak intensities occurred at about 440 nm, whereas the maximal intensity in the sample extracts occurred at 450nm.

### Phenolic content: Comparison of methods

**Figure 3:**
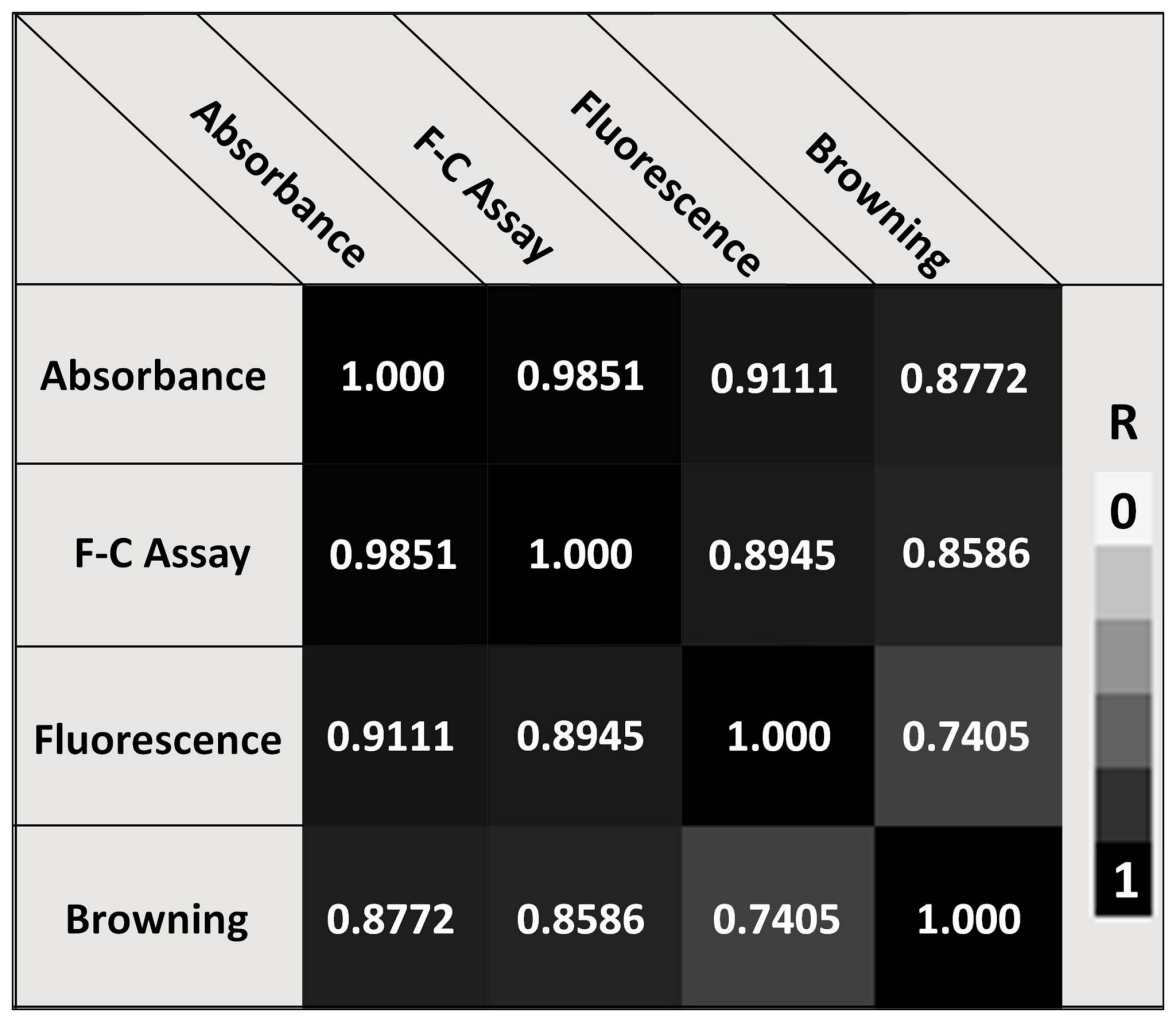
Correlation values comparing various analytical methods with one another and visual tissue browning scores. Analytical methods were conducted on solvent extracts of *Artemisia annua* callus grown on various media and include a modified Folin-Coicalteu methods (F-C Assay), absorbance at 340 nm (absorbance), and autofluorescence (excitation at 360 nm, emissions at 450 nm; Fluorescence).

The Folin-Coicalteu assay, absorbance at 340nm, fluorescence intensity readings, and visual tissue browning scores were in general agreement but there were some discrepancies (Table 1; Figure 3). In all cases, tissue grown in the presence of AIP produced significantly lower values than tissue grown in its absence. Callus grown on 2,4-D medium had similar visual browning scores and phenolic content (based on the Folin assay) as BA/NAA grown callus. However, extracts from BA/NAA grown callus had significantly higher absorbance at 340 nm and produced significantly higher emissions at 460 nm (360 nm excitation) than extracts from 2,4-D grown callus. The results from all four methods significantly correlated with one another with r values ranging from a low of 0.7259 between fluorescence and browning scores to a high of 0.9851 between absorbance measurements and the F-C assay (Figure 3). The correlation values were higher when the analysis was run on 2,4-D media or BA/NAA media separately (data not shown). Regression analysis comparing each method to visual tissue browning resulted in significant relationships for all three methods. The coefficients of determination were 0.7570, 0.7398, and 0.5270 between tissue browning scores and results from absorbance (340 nm), the Folin-Colcalteu assay, and fluorescence intensity, respectively. However, when the regressions were run separately for 2, 4-D and BA/NAA based media the coefficients of determination were greater. For 2,4-D media, they were 0.7924, .7899, and 0.5815 for absorbance (340 nm), the F-C assay, and fluorescence intensity, respectively. The values from callus grown on BA/NAA media were 0.9463, 0.9374, and 0.8417 for absorbance (340 nm), the F-C assay, and fluorescence intensity, respectively.

### Fluorescence – Microscopic evaluation

**Figure 4:**
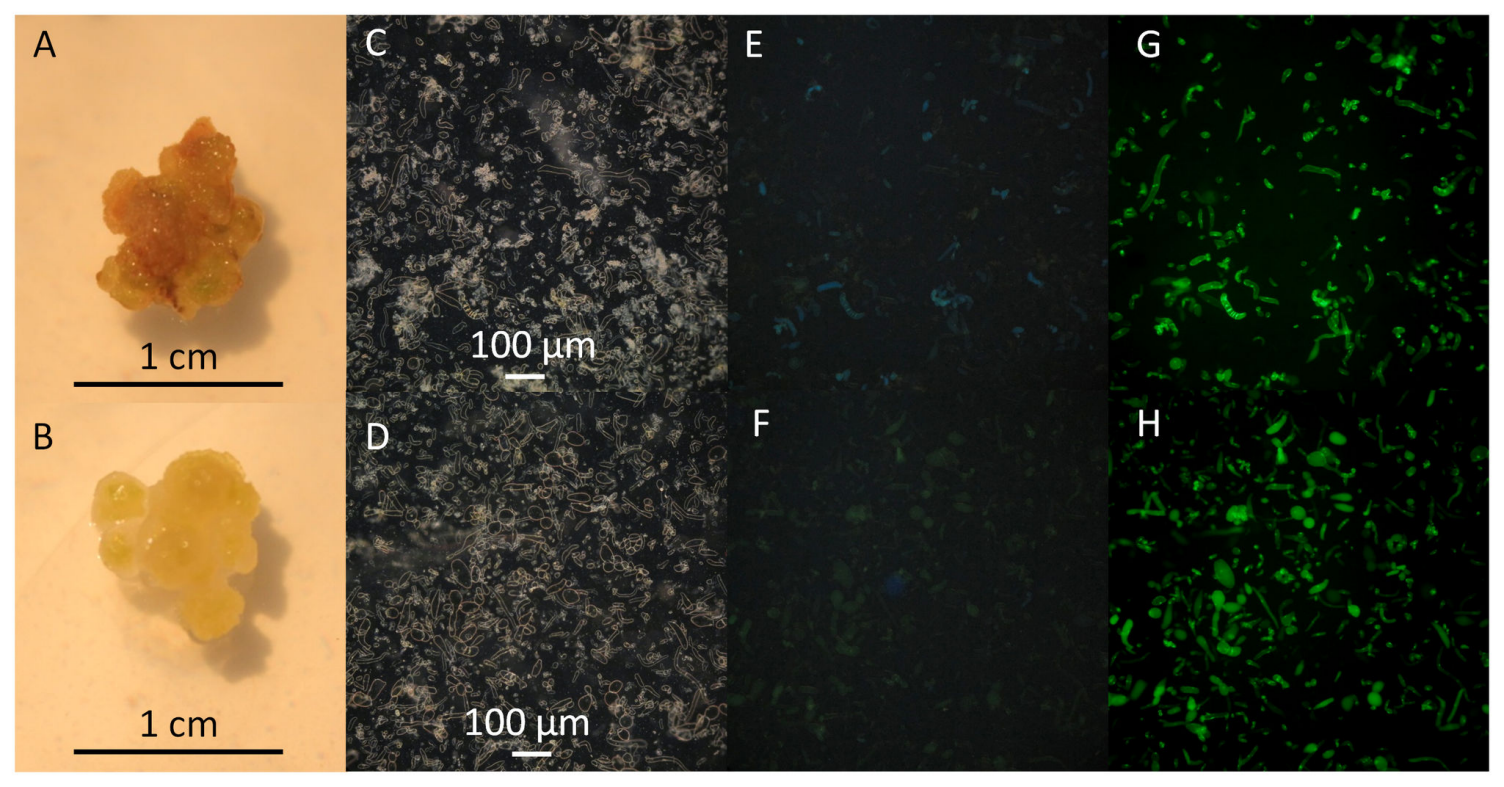
Macroscopic and microscopic observations of *Artemisia annua* callus grown on MS medium supplemented with 4.5 µM 2,4-D (A,C,E,G) and the same medium with 100 µM AIP incorporated (B,D,F,H). A-B: callus at a macroscopic level, C-D: callus cells using brightfield microscopy with phase contrast, E-F: UV induced fluorescence of callus cells stained with fluorescein diacetate (blue=autofluorescence, green=stain), and G-H: cell viability using fluorescein diacetate.

**Figure 5:**
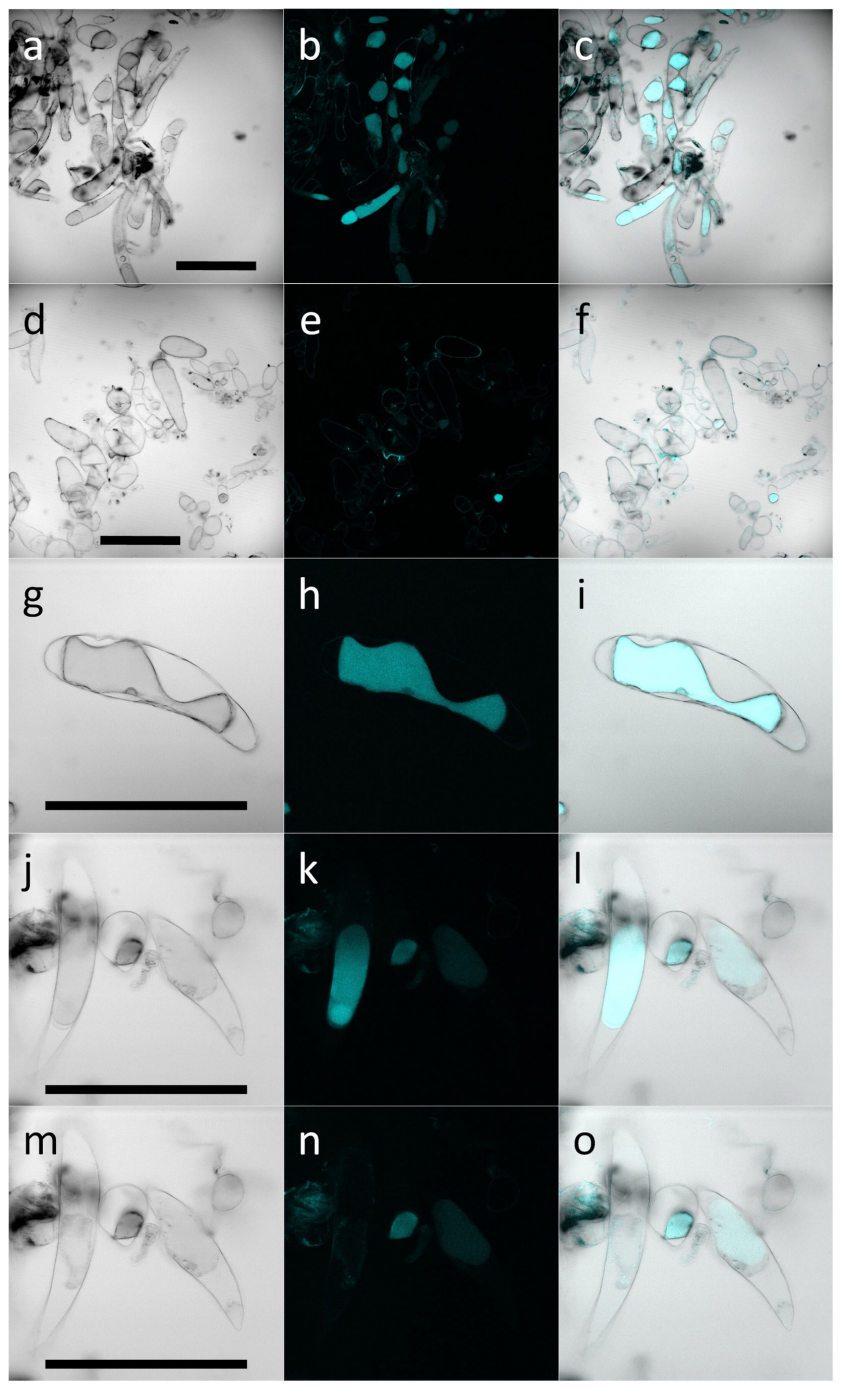
Brightfield (a,d,g,j,m), confocal (b,e,h,k,n), and overlays (c,f,i,l,o) of *Artemisia annua* callus cells cultured on MS based media supplemented with 4.5 µM 2,4-D (a-c), and MS based media supplemented with 4.5 µM 2,4-D and 100 µM AIP (d-f). Images g-i depict an autofluorescent plasmolyzed cell cultured on MS based media supplemented with 4.5 µM 2,4-D. Panels j-o show autofluorescent plasmolyzed cells cultured on MS based media supplemented with 4.5 µM 2,4-D before (j-l) and after (m-o) cell rupture. Confocal images were obtained using 405 nm excitation and emissions between 430-480 nm. Scale bars represent µm.

Blue-green auto-fluorescence in callus grown on 2,4-D media with or without 100 µM AIP was observed using an epi-fluorescence inverted microscope using UV excitation (Figure 4) as well as a confocal microscope using a 405 nm laser (Figure 5). Fluorescence was observed in callus grown on either media, but was much more abundant in callus grown in the absence of AIP. Callus grown on both media stained for viability with fluorescein diacetate (FDA), but the proportion of cells that stained appeared to be greater in callus grown on AIP (Figure 4); however, this was not quantified due to the heterogeneity of the tissue. In general, callus grown on basal 2,4-D medium exhibited browning concentrated in the region where the tissue was in direct contact with the medium and this region of the tissue did not stain for viability with FDA. Upon closer inspection, it was found that the blue-green auto-fluorescent material was primarily found within plasmolyzed cells, but could also be observed to a lesser extent in some cell walls. During microscopic observation, plasmolyzed cells often burst and fluorescent material dissipated into the surrounding liquid (Figure 5).

### Efficacy of AIP for reducing browning in other species

**Figure 6:**
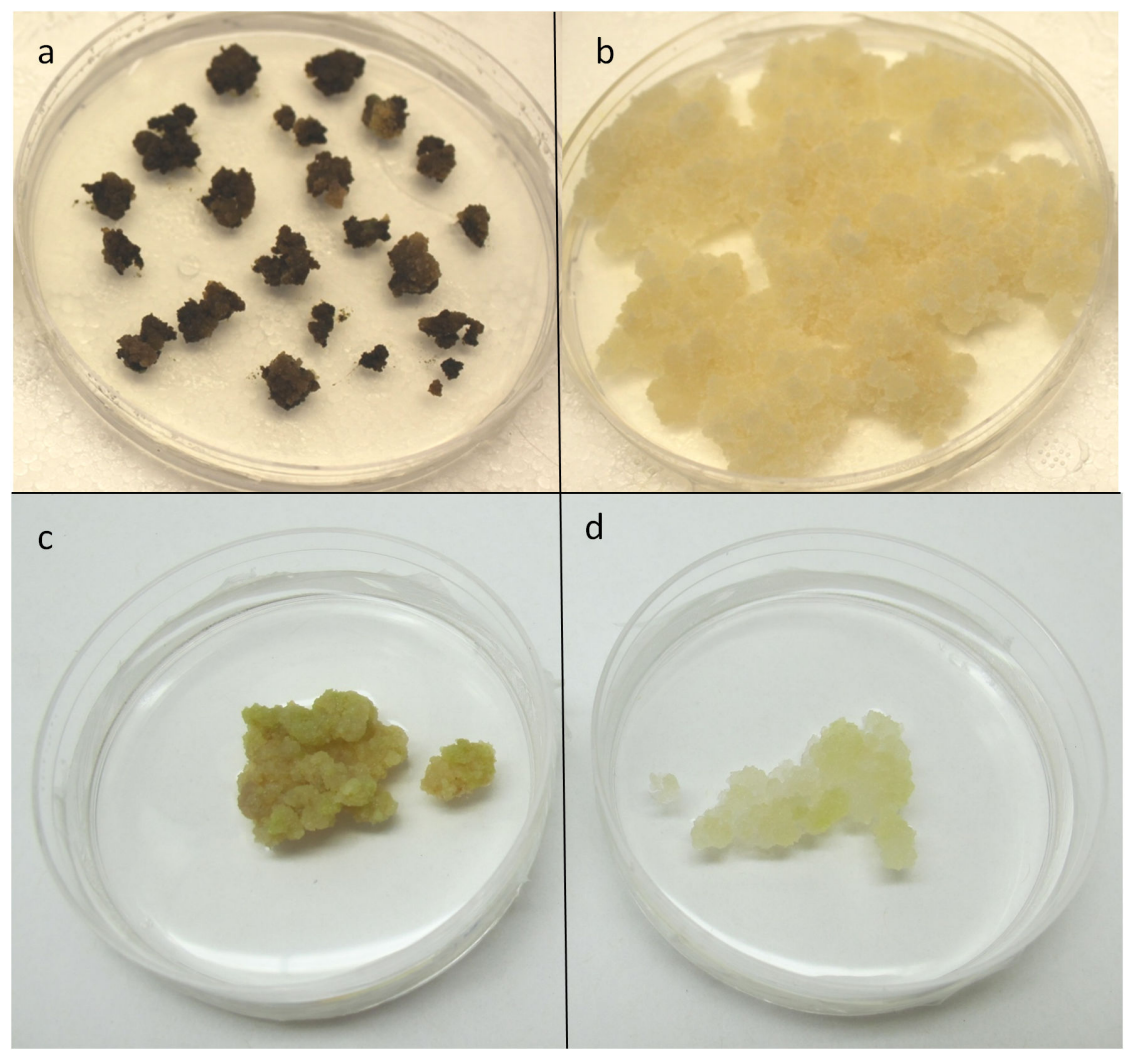
American elm (a and b) and sugar maple (c and d) callus cultured on MS media supplemented with 5 µM BA and 1 µM NAA with (b and d) or without (a and c) the addition of 1 mM AIP.

Callus cultures of sugar maple (*Acer saccharum*) and American elm (*Ulmus americana*) cultured on basal media both exhibited tissue browning to varying degrees with *U. americana* being more prone to this problem (Figure 6). In both cases, tissue browning was significantly reduced when cultured on the same medium supplemented with 1mM AIP (Figure 6)

## Discussion

This study reports a novel and highly effective approach to reduce oxidative browning in plant tissue culture by inhibiting the PAL enzyme with the competitive inhibitor 2-aminoindane-2-phosphonioc acid (AIP). Incorporation of AIP into the media resulted in a significant and dramatic reduction in visible browning (Figure 4; Table 1). A similar reduction of browning in the presence of AIP [35], although not quantified, has previously been observed in callus of American elm (*Ulmus americana*), a species that exhibits extensive callus browning [19]. The reduction in browning observed in the current study was accompanied by a general increase in callus growth, with a two-fold increase on BA/NAA based medium. The increase in callus growth is presumably a consequence of reducing the toxic effects associated with oxidative browning; however, inhibition of this pathway may also cause a re-allocation of carbon resources [44] or interact with endogenous plant growth regulators, which could have contributed to the increased growth.

While tissue browning is a visible characteristic that can be assessed using a hedonic scale, this approach is relatively subjective and makes definitive comparisons difficult, especially among independent studies. Visual browning observations are often further complicated by the presence of chlorophyll that mask the brown pigmentation as with the BA/NAA generated callus in the current study. As such, the following alternative methods to more objectively measure the efficacy of AIP were evaluated: a modified Folin-Ciocalteu (F-C) method [42], absorbance at 340nm [34], and phenolic based auto-fluorescence [43]. The three methods were in general agreement with one another as well as the visual browning scores, suggesting that they may be useful approaches to objectively measure tissue browning. Based on regression analyses, the best method to predict tissue browning was absorbance at 340 nm, with a coefficient of determination of 0.7570. This method has previously been used as a proxy to measure post harvest browning in lettuce, where it was also found to be in agreement with visual scores as well as the hue angle [34]. The co-efficient of determination increased to 0.7924 when the analysis was conducted on callus from the 2,4-D based media separately and 0.9463 when comparing just among BA/NAA grown callus, suggesting that visual browning scores were more consistent within each callus type.

The three methods evaluated rely on direct or indirect measurements of compounds derived from the phenylpropanoid pathway. However, because of the diversity of phenolic compounds there is no single method that can accurately quantify all of them. The widely used F-C assay indirectly estimates total phenols based on their ability to reduce phos-phomolybdic /phosphotungstic acid complexes, causing a shift in their absorptive properties that can be determined by measuring absorbance [42,45]. However, phenolic compounds differ in their reducing properties such that the chemical composition can alter the response in this assay. Further, a number of non-phenolic compounds such as ascorbic acid can also elicit the reaction and thereby interfere with the assay [45,46]. In some cases, the F-C assay under-reports the phenolic content compared to other methods while in other cases it can overestimate them due to interfering compounds [46,47].

Absorbance at 340 nm directly measures phenolics based upon their absorptive profiles. As with the F-C assay, the property of each phenolic compound is unique and the composition of phenolic compounds in the sample, as well as the standards that are employed, will influence the estimate. For example, the estimated phenol content of callus grown on 2,4-D medium with 100 µM AIP was 75% higher using the standard curve generated by ferulic acid compared to that of chlorogenic acid. Additionally, like the F-C assay, there is the potential for common non-phenolic compounds such as chlorophyll and carotenoids to interfere with the assay. While this approach has successfully been employed with lettuce tissue [34] and appears to be suitable to estimate tissue browning in *Artemisia annua* callus, it would likely be unsuitable in many plant systems where carotenoids or chlorophyll are more abundant.

The third method evaluated in this study relies on the fluorescent properties of phenolic compounds. Phenolic compounds, including lignin, are responsible for the blue green emissions of plant material when excited with UV light [43]. However, not all phenolic compounds exhibit this property, and as a result this approach can only detect a subset of phenolic compounds. Further, in the current study, ferulic and chlorogenic acid fluorescence exhibited a non-linear relationship that saturated at relatively low concentrations. In contrast, a dilution series of the most fluorescent sample extract behaved in a linear fashion that did not saturate and had peak intensities approximately 10 nm different than the standards. Together, these data suggest that ferulic and chlorogenic acid monomers were not the compounds responsible for the fluorescence of the extracts and that they are not suitable as standards in this system. However, given that the dilution series of the sample extract behaved in a linear fashion, this method appears to be suitable for relative comparisons among samples.

The potential value of measuring the fluorescence of the extract was demonstrated by fluorescence microscopy of the tissues. The blue-green autofluorescence corresponding to spectra produced from the sample extracts (Figure 1) was highly localized within single cells rather than being evenly distributed (Figures 4 & 5). Upon closer observation, the cells that contained the fluorescent material were highly plasmolyzed (Figure 5). While some of the autofluorescent cells stained for viability with FDA (Figure 4), they were commonly observed to rupture and release the fluorescent material during observation. As such, it appears that these cells may have been undergoing a process of controlled cell death, perhaps representing a defense response akin to the hypersensitive response [9]. Phenolic compounds are an important mechanism related to programmed cell death in plants, especially in regard to defense responses [48,49]. The hypersensitive response is generally regulated by salicylic acid, a product of the phenylpropanoid pathway [49,50]. If the plasmolyzed autofluorescent cells observed in the current study were indeed a form of programmed cell death, AIP may reduce its occurrence by preventing the synthesis of the signalling molecule and/or by preventing the ability of cells to accumulate phenolic compounds. Regardless, the accumulation and rupture of these cells provides a mechanism in which large amounts of phenolic compounds are released into the surrounding tissue and growth medium that is inhibited by the addition of AIP. The relative fluorescence of the samples appears to be related to the number of cells that have gone through this process and could provide a valuable tool for investigating tissue browning.

The current study demonstrates the efficacy of inhibiting the phenylpropanoid pathway with AIP as a novel preventative approach to control tissue browning in *A. annua*. Preliminary experiments with sugar maple and American elm demonstrate that this approach is also effective in these species, suggesting the efficacy of this approach to reduce tissue browning in a wide variety of plants. More work is needed to determine optimal concentrations of AIP, assess the potential of combining this approach with other methods, and evaluate the effects that inhibiting this pathway may have on plant growth, development, and regeneration. Regardless, inhibiting the production of phenolic compounds with the incorporation of AIP may greatly improve our ability to prevent tissue browning and expand the application of tissue culture techniques to species that are currently recalcitrant to in vitro manipulation.
